# RACIAL DISCRIMINATION TRAJECTORIES AND MULTIMORBIDITY BURDEN AND CHANGE AMONG MIDDLE-AGED AND OLDER BLACKS

**DOI:** 10.1093/geroni/igad104.3550

**Published:** 2023-12-21

**Authors:** Kellee White Whilby, Kaitlynn Robinson-Ector, Ana Quiñones

**Affiliations:** University of Maryland, College Park, Maryland, United States; University of Maryland, College Park, Maryland, United States; Oregon Health & Science Institute, Portland, Oregon, United States

## Abstract

Racial discrimination is a chronic psychosocial stressor that can accelerate aging. Characterizing longitudinal patterns of racial discrimination may provide greater precision in predicting health risk particularly for outcomes such as multimorbidity that is considered a proxy of multisystem dysregulation and a marker of cumulative disease burden. However, longitudinal patterns of racial discrimination and its relationship with multimorbidity has received limited attention. We investigate the association between racial discrimination trajectories (RDT) and multimorbidity among a subsample of middle-aged and older Black adults from the Health and Retirement Study (2006–2020, N = 2,167, 50+). RDTs were constructed based on the Everyday Discrimination Scale using repeated measures latent profile analyses. Multimorbidity was operationalized using the multimorbidity weighted index (MWI) score. Linear mixed models, adjusted for sociodemographic, behavioral, and clinical covariates, estimated the association between RDT and MWI score and change in score over time. Average MWI scores varied across RDT classes: low (5.9), moderate (6.5), and high (5.9). Individuals characterized in the moderate RDT had the highest multimorbidity burden (3.03; 95% confidence interval [CI]: 1.46, 4.58) and was the only group that experienced an accelerated rate of multimorbidity accumulation, as measured by change in MWI score over time. This relationship was not observed among respondents in the persistently high RDT. Our findings suggest that there are differences in the relationship between RDT and multimorbidity burden. Uncovering specific characteristics associated with the profiles of RDT may advance our understanding of healthy aging processes.

